# Pattern-based clinical recognition of diabetes-associated *mucormycosis:* an evidence mapping study integrating symptom clustering and diagnostic pathways

**DOI:** 10.3389/fcimb.2026.1861349

**Published:** 2026-07-03

**Authors:** Qiongfang Zhang, Ze Fang, Hailing Zeng, Yiyang Gong

**Affiliations:** Zhongjiang People’s Hospital, Zhongjiang, China

**Keywords:** diabetes mellitus, diagnostic pathway, evidence mapping, mucormycosis, pattern-based recognition, rhino-orbito-cerebral mucormycosis, symptom clustering

## Abstract

**Background:**

Diabetic-associated *mucormycosis* is a rapidly progressive and life-threatening opportunistic infection with high mortality. Early recognition remains challenging due to heterogeneous clinical presentations and the lack of structured characterization of symptom patterns. This study aimed to systematically map clinical features and develop a pattern-based recognition framework for diabetic-associated *mucormycosis*.

**Methods:**

A comprehensive literature search was conducted in PubMed, Web of Science, and Embase from database inception to March 2026. Case reports and case series involving diabetic patients with confirmed *mucormycosis* were included. Case-level data were extracted and analyzed using an evidence mapping approach. Clinical presentation clusters were defined based on infection sites, and symptom distributions, imaging features, and diagnostic pathways were descriptively synthesized.

**Results:**

A total of 66 studies comprising 97 cases were included. Rhino-orbito-cerebral *mucormycosis* (ROCM) was the predominant presentation (68.0%), followed by pulmonary infection (14.4%). Symptom patterns demonstrated a distinct structure characterized by intra-cluster aggregation and inter-cluster separation. ROCM was associated with a concentrated pattern of visual and craniofacial manifestations, whereas pulmonary cases were dominated by respiratory symptoms. Importantly, diagnostic pathways exhibited a symptom-driven structure, in which clinical presentation guided sampling strategies and influenced diagnostic methods. Based on these findings, a structured framework linking presentation clusters, symptom combinations, and diagnostic pathways was established.

**Conclusions:**

Diabetic-associated *mucormycosis* appears to exhibit relatively structured clinical presentation patterns within the published literature. The proposed pattern-based recognition framework provides a descriptive and hypothesis-generating synthesis of clinical presentation patterns and diagnostic pathways in diabetic-associated mucormycosis. This framework may help inform future research and contribute to a more structured understanding of clinical recognition patterns in high-risk populations.

## 1 Introduction

### 1.1 Epidemiology and clinical burden of *mucormycosis*

*Mucormycosis* is an invasive opportunistic infection caused by fungi of the order Mucorales. Although its overall incidence remains relatively low, a gradual increase has been observed in recent years, particularly among immunocompromised populations (Turunc et al., 2008; Diongue et al., 2025). The disease is widely distributed worldwide and is primarily acquired through inhalation of airborne spores or direct inoculation via cutaneous wounds. Under susceptible host conditions, it can rapidly progress to an invasive disease (Turunc et al., 2008; Mohsin et al., 2024).

Depending on the site of infection, *mucormycosis* may present in various clinical forms, including rhino-orbito-cerebral, pulmonary, gastrointestinal, cutaneous, and disseminated types. Among these, rhino-orbito-cerebral *mucormycosis*(ROCM) is the most common clinical presentation and occurs predominantly in patients with diabetes mellitus (Anane et al., 2009).

*Mucormycosis* is characterized by rapid progression and high invasiveness. Its pathophysiological hallmark is angioinvasion, which can lead to thrombosis, tissue ischemia, and extensive necrosis, often resulting in a fulminant clinical course (Eker et al., 2023). Even with aggressive antifungal therapy and surgical intervention, the prognosis remains poor. Previous studies have reported an overall mortality rate of up to 50% or higher, which may reach 75%–95% in cases of disseminated infection (Turunc et al., 2008). For patients with ROCM, the mortality rate is typically around 50%–60% (Eker et al., 2023).

In addition to high mortality, the disease is associated with severe disabling sequelae, including vision loss, orbital exenteration, and central nervous system involvement, all of which significantly impair patients’ quality of life (Abdolalizadeh et al., 2019).

In recent years, the population at high risk for *mucormycosis* has continued to expand, driven by the rising prevalence of diabetes mellitus, the widespread use of immunosuppressive therapies, and emerging factors such as COVID-19. Recent expert consensus statements have further emphasized the growing global burden of secondary mucormycosis following respiratory viral infections, particularly among patients with diabetes mellitus, corticosteroid exposure, and immunometabolic dysfunction. Consequently, the overall disease burden has shown an increasing trend (Abdolalizadeh et al., 2019; Sonpal et al., 2024; Qu et al., 2025; Yelavarthy et al., 2025). Particularly in developing countries and regions with a high prevalence of diabetes, ROCM has emerged as one of the most representative forms of invasive fungal infections, posing a substantial challenge to clinical diagnosis and management.

### 1.2 Biological mechanisms of diabetes as a high-risk host

Diabetes mellitus, particularly in the presence of poor glycemic control or diabetic ketoacidosis (DKA), is considered one of the most important host-related risk factors for *mucormycosis* (Abdolalizadeh et al., 2019). Among patients with rhino-orbito-cerebral *mucormycosis* (ROCM), the prevalence of diabetes is markedly high, highlighting its critical role in disease pathogenesis (Anane et al., 2009). Recent public health analyses have further emphasized the growing burden of invasive fungal infections in high-risk populations, particularly among patients with diabetes mellitus and other forms of immunometabolic dysfunction (Miao et al., 2026).

The increased susceptibility is primarily attributed to the synergistic effects of impaired immune function and alterations in the metabolic environment. Hyperglycemia can impair neutrophil chemotaxis, phagocytosis, and intracellular killing, while DKA further promotes the release of free iron in serum, thereby creating a favorable environment for fungal growth and invasion (Turunc et al., 2008).

In addition, *mucormycosis* is characterized by pronounced angioinvasion. Diabetes-associated microangiopathy may exacerbate local ischemia and tissue necrosis, facilitating the spread of infection to the orbit and intracranial structures (Eker et al., 2023).

Clinical studies have also demonstrated that patients with hyperglycemia or DKA are more likely to develop severe complications and experience poor clinical outcomes (Gulmez et al., 2025). Therefore, diabetes significantly increases the risk of *mucormycosis* and accelerates disease progression through the dual mechanisms of immune suppression and metabolic dysregulation.

### 1.3 Clinical heterogeneity and challenges in early recognition

The clinical manifestations of *mucormycosis* are highly heterogeneous, with the symptom spectrum largely depending on the site of infection, underlying comorbidities, and stage of disease progression. Rhino-orbito-cerebral *mucormycosis*(ROCM) typically originates in the paranasal sinuses and may initially present with nonspecific symptoms such as fever, headache, nasal congestion, or facial pain. It can subsequently progress rapidly to orbital involvement (e.g., proptosis, ophthalmoplegia, and vision loss) and even intracranial extension (Turunc et al., 2008).

In some patients, the disease may present with atypical or insidious onset, occasionally lacking classical features such as DKA or overt signs of infection, which further complicates early clinical recognition (Turunc et al., 2008).

There is considerable inter-patient variability in clinical presentation, including differences in symptom combinations, progression rates, and disease severity. Manifestations may range from localized sinusitis to severe forms characterized by angioinvasion, extensive tissue necrosis, and central nervous system involvement (Anane et al., 2009). This heterogeneity makes it difficult for clinicians to rely on a single or typical symptom for early diagnosis.

More importantly, *mucormycosis* progresses rapidly within a narrow therapeutic window, and delayed diagnosis has been identified as a major contributor to its high mortality (Anane et al., 2009). Existing evidence indicates that the interval between symptom onset and definitive diagnosis often ranges from several days to weeks. During this period, the infection may rapidly extend to critical anatomical structures, significantly increasing the risks of mortality and disability.

This challenge is particularly pronounced in patients with diabetes mellitus. Therefore, there is an urgent need to systematically integrate existing evidence to better delineate the clinical presentation spectrum and to optimize strategies for early recognition.

### 1.4 Limitations of existing evidence, knowledge gaps, and study objectives

Although previous studies have reported certain clinical features of diabetic-associated *mucormycosis*, the current body of evidence remains subject to substantial limitations. Most available studies are single-center retrospective case series with relatively small sample sizes, and the study populations are predominantly focused on rhino-orbito-cerebral *mucormycosis*(ROCM). As a result, clinical manifestations across different infection sites, host conditions, and stages of disease progression have not been systematically integrated (Abdolalizadeh et al., 2019).

Moreover, existing literature has largely emphasized mortality, surgical interventions, and prognostic outcomes, whereas clinical features relevant to early recognition—such as symptoms, signs, imaging findings, and laboratory abnormalities—remain fragmented and insufficiently synthesized. Consequently, a comprehensive clinical presentation spectrum tailored for early diagnosis has yet to be established (Gulmez et al., 2025).

In addition, a subset of patients may present with atypical or non-classical features, including the absence of characteristic risk indicators, further complicating the interpretation of existing evidence and its application in clinical practice (Turunc et al., 2008).

For patients with diabetes mellitus, unique host susceptibility mechanisms, patterns of organ involvement, and disease progression trajectories may exist. However, to date, there is a lack of evidence mapping studies specifically focused on this population that systematically characterize clinical recognition features and the distribution of presentation phenotypes.

Therefore, this study aimed to employ an evidence mapping approach to systematically synthesize and visually present the clinical recognition features, anatomical distribution, and phenotypic spectrum of diabetic-associated *mucormycosis*. By doing so, we sought to identify patterns in the existing evidence, highlight current knowledge gaps, and provide a structured framework to support early clinical recognition and inform future research design.

## 2 Methods

### 2.1 Study design

This study was designed as an evidence mapping analysis aimed at systematically integrating case-level data from published literature on diabetic-associated *mucormycosis*, in order to characterize its clinical recognition features and the distribution of presentation phenotypes.

The study design was informed by established methodological frameworks for systematic reviews and scoping reviews. The conduct and reporting of this study adhered to the Preferred Reporting Items for Systematic Reviews and Scoping Reviews (PRISMA-ScR) guidelines.

### 2.2 Data sources and search strategy

A comprehensive literature search was conducted in PubMed, Web of Science, and Embase databases to identify relevant studies. The search covered the period from database inception to March 2026.

The search strategy combined controlled vocabulary terms (Medical Subject Headings [MeSH] and Emtree terms) with free-text keywords, including “*mucormycosis*,” “zygomycosis,” “diabetes mellitus,” and “diabetic ketoacidosis.” The search strategy was adapted as appropriate for each database. The full search strategy is provided in **Supplementary Table 1**.

In addition, the reference lists of all included studies were manually screened to identify any potentially eligible articles and minimize the risk of missing relevant studies. No language restrictions were applied in this study.

### 2.3 Study selection and eligibility criteria

The study selection process was conducted independently by two reviewers. An initial screening was performed based on titles and abstracts, followed by full-text assessment of potentially eligible studies. Any disagreements were resolved through discussion and, if necessary, adjudicated by a third reviewer. Inter-reviewer agreement for study selection was assessed using Cohen’s kappa coefficient, demonstrating excellent agreement between the two reviewers (κ = 0.86).

#### 2.3.1 Inclusion criteria

Studies were included if they met the following criteria:

1. The study population consisted of patients with diabetes mellitus.
2. A confirmed diagnosis of *mucormycosis* was established, based on histopathology, fungal culture, molecular testing, or clearly documented diagnostic criteria in the original report.
3. The study reported at least one clinically relevant feature related to disease recognition, including symptoms, signs, site of infection, clinical syndrome classification, or imaging findings, and provided sufficient information to assign cases to predefined clinical presentation clusters.
4. The study design was a case report or case series.

#### 2.3.2 Exclusion criteria

Studies were excluded if they met any of the following criteria:

1. The study population did not include patients with diabetes mellitus.
2. Clinical presentation or infection site information was not reported.
3. The infection was not *mucormycosis*.
4. Experimental studies (including animal or *in vitro* studies).
5. Reviews, commentaries, or editorials.
6. Duplicate case reports.

### 2.4 Data extraction

Data extraction was performed independently by two reviewers and subsequently cross-checked for accuracy. Inter-reviewer agreement for data extraction also demonstrated high consistency (κ = 0.84). Any discrepancies were resolved through discussion until consensus was reached.

Case-level data were extracted from each included study and organized into a structured database. The extracted variables were categorized as follows:

1. Host characteristics: age, sex, type of diabetes mellitus, and the presence of diabetic ketoacidosis (DKA).
2. Clinical manifestations: site of infection, classification of clinical presentation clusters, and major symptoms (recorded as keywords).
3. Imaging findings: including, but not limited to, sinus involvement, orbital invasion, intracranial involvement, pulmonary consolidation, or cavitation.
4. pathway: sampling site and diagnostic methods (e.g., histopathology, fungal culture, or molecular testing).
5. Outcome data: surgical intervention, antifungal therapy, and survival outcomes.

For variables that were not explicitly reported in the original studies, the data were recorded as missing and no imputation or assumptions were made.

### 2.5 Data synthesis and evidence mapping analysis

A descriptive analytical approach was employed to systematically synthesize the included cases. First, the number of cases within each clinical presentation cluster was quantified to characterize their distribution patterns. Second, frequency analyses of symptoms and imaging findings were conducted within each cluster to identify predominant symptom combinations and potential clustering patterns. Third, by integrating information on sampling sites and diagnostic methods, a structured diagnostic framework linking “clinical presentation–sampling site–diagnostic method” was constructed.

Furthermore, differences in symptom composition across clinical presentation clusters were compared to explore potential features relevant to clinical recognition. Associations between host factors (e.g., diabetic ketoacidosis [DKA]) and the distribution of clinical presentation clusters were also examined using descriptive analysis. All analyses were descriptive in nature, and no statistical inference was performed.

For evidence presentation, an evidence mapping approach was applied to visually illustrate the structural relationships among clinical presentations, diagnostic pathways, and host factors. Data management and visualization were conducted using R software (R Foundation for Statistical Computing, Vienna, Austria).

Given that this study was designed as an evidence mapping analysis aimed at describing evidence distribution and identifying patterns, rather than estimating effect sizes or establishing causal relationships, no protocol registration was performed.

## 3 Result

### 3.1 Study selection and case identification

A total of 4,248 records were identified through database searching. After removal of duplicates, 2,406 records remained for title and abstract screening, of which 2,309 were excluded.

Subsequently, 97 articles were assessed for full-text eligibility. Among these, 15 studies were excluded due to the unavailability of full texts, resulting in 82 articles proceeding to eligibility assessment.

Following further screening, studies with duplicate case reporting or lacking clinical presentation–related information were excluded. Ultimately, 66 studies (all case reports or case series) were included for case-level analysis (see References Alemayehu et al., 2022; Ali Asghar et al., 2019; Aljoaid et al., 2023; Alqhamdi et al., 2019; Al Saad et al., 2021; Alsaeedi et al., 2022; Alsayed et al., 2024; Arora et al., 2023; Awan et al., 2024; Biradar, 2016; Chadli-Chaieb et al., 2005; Chander et al., 2011; Das et al., 2024; Dökmetaş et al., 2002; Erami et al., 2013; Eslamian et al., 2022; Fattah et al., 2018; Fouzi et al., 2009; Galletti et al., 2020; Göktürk, 2025; Harrar et al., 2024; Hsiao et al., 2002; Huddle et al., 1987; İnal et al., 2022; Jawad et al., 2024; Jawanda et al., 2021; Jawanda et al., 2021; Kabir, 2025; Katsitadze et al., 2024; Kaur et al., 2022; Kaushal et al., 2022; Khandelwal et al., 2012; Lädrach et al., 2024; Lee et al., 2004; Lee et al., 1996; Luo et al., 2022; Maduranga et al., 2025; Manji et al., 2019; Martín, et al., 2017; Martínez-Herrera et al., 2020; Modarelli et al., 2023; Mohammadi et al., 2015; Monroig and Tarquinio, 2022; Munhoz et al., 2022; Nair et al., 2021; Nolan et al., 1989; Oliveira and Milech, 2002; Ortega et al., 2022; Pennell et al., 2018; R et al., 2020; Randhawa et al., 2021; Reich et al., 1985; Shahanikelaki et al., 2023; Shahzadi et al., 2024; Shekar et al., 2015; Simijiang et al., 2025; Sumual et al., 2024; Tsisar et al., 2025; Waghmare et al., 2022; Wang et al., 2023; West et al., 1995; Zayet et al., 2021).

**Figure 1:**
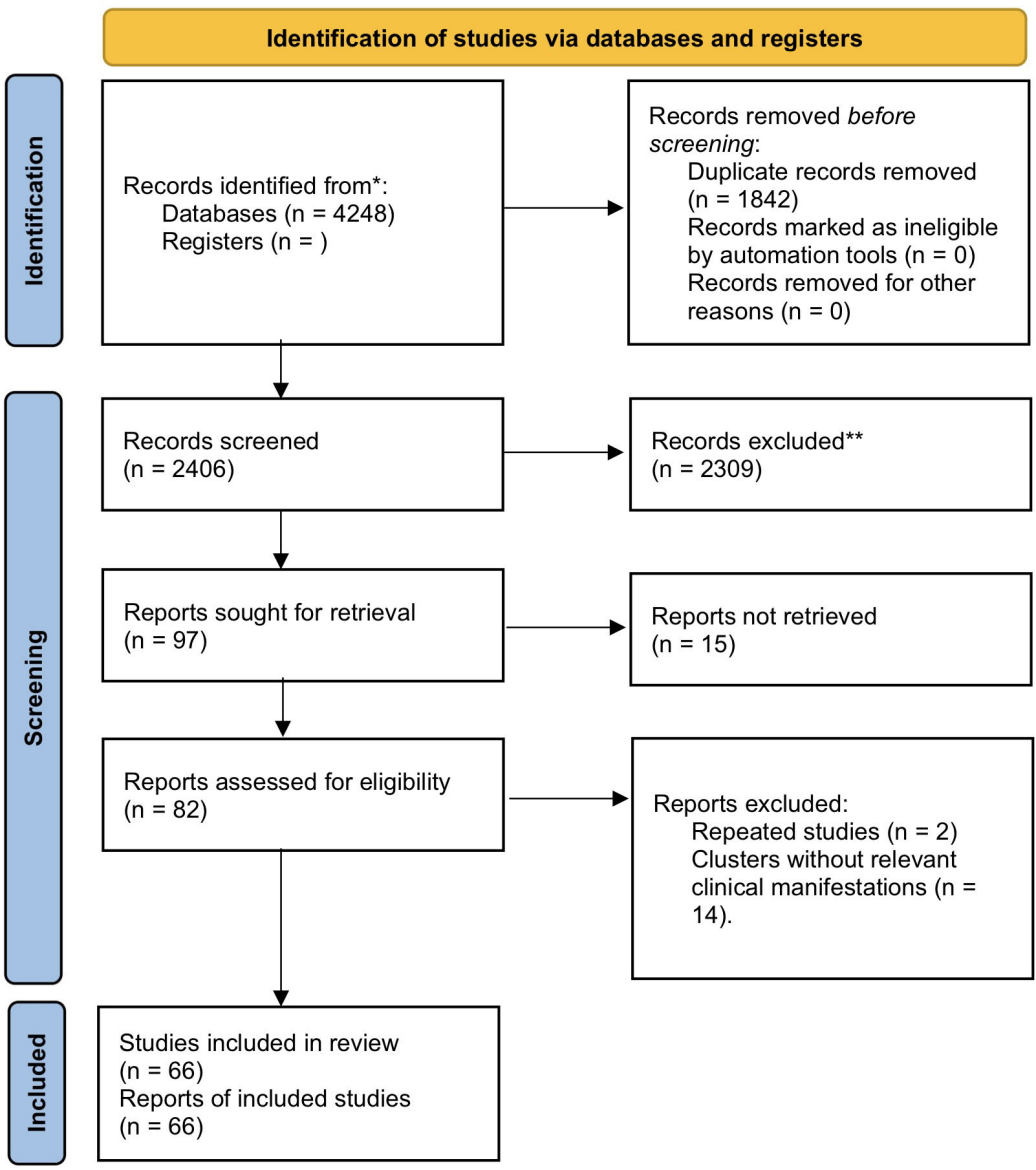
Flow diagram of study selection and case inclusion.

The detailed study selection process is illustrated in **Figure 1**.

### 3.2 Baseline characteristics of included cases

A total of 97 cases were included in the case-level analysis (**Table 1**). Age information was available for 96 cases, with a range of 6–75 years, a mean age of 45.52 years, and a median age of 47 years.

**Table 1:** Baseline characteristics of included cases.

Variable	Value
Total cases	97
Age, years, mean ± SD	45.52 ± 17.47
Sex, n	Male: 59; Female: 35; Not reported: 3
Diabetes type, n	T1DM: 18; T2DM: 39; Not specified: 40
Diabetic ketoacidosis, n	Present: 36; Absent: 13; Not specified: 48

Regarding sex distribution, 59 cases were male and 35 were female, while sex was not reported in 3 cases, indicating a slight male predominance. In terms of diabetes type, 39 cases were classified as type 2 diabetes mellitus (T2DM) and 18 as type 1 diabetes mellitus (T1DM), whereas 40 cases did not specify the diabetes subtype.

Diabetic ketoacidosis (DKA) was reported as present in 36 cases and absent in 13 cases, while 48 cases did not report DKA status, indicating a relatively high proportion of missing data for this variable in the existing literature.

Overall, diabetic-associated *mucormycosis* occurred across a wide age range, predominantly affecting adults. Host-related variables, such as diabetes subtype and DKA status, exhibited a certain degree of heterogeneity in reporting across studies. Detailed case-level data are provided in **Supplementary Table 2**.

### 3.3 Distribution of clinical presentation clusters

**Figure 2:**
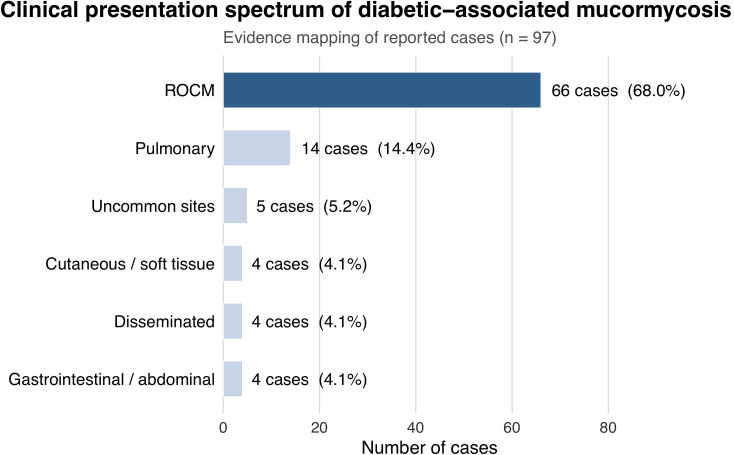
Distribution of clinical presentation clusters in diabetic-associated *mucormycosis.*.

The included cases were categorized into clinical presentation clusters based on the site and extent of infection (**Figure 2**). The distribution of these clusters demonstrated a markedly uneven pattern. Rhino-orbito-cerebral *mucormycosis* (ROCM) was overwhelmingly predominant, accounting for 66 cases (68.0%), with a proportion exceeding that of all other types combined. Pulmonary infection ranked second, comprising 14 cases (14.4%), although a substantial gap remained compared with ROCM.

The remaining clinical types were relatively infrequent and more evenly distributed, including uncommon-site infections (5 cases, 5.2%), cutaneous and soft tissue infections (4 cases, 4.1%), disseminated infections (4 cases, 4.1%), and gastrointestinal/abdominal infections (4 cases, 4.1%). No clear dominance was observed among these less frequent categories.

Overall, diabetic-associated *mucormycosis* exhibited a characteristic “single-dominant” distribution pattern, in which a high-frequency core cluster (ROCM) was distinctly stratified from multiple low-frequency categories. This distribution pattern suggests a pronounced anatomical predilection in diabetic hosts, with clinical manifestations concentrated in specific anatomical regions rather than being uniformly distributed. Among these, ROCM represents the primary contributor to the overall disease burden.

### 3.4 Symptom characteristics across clinical presentation clusters

**Figure 3:**
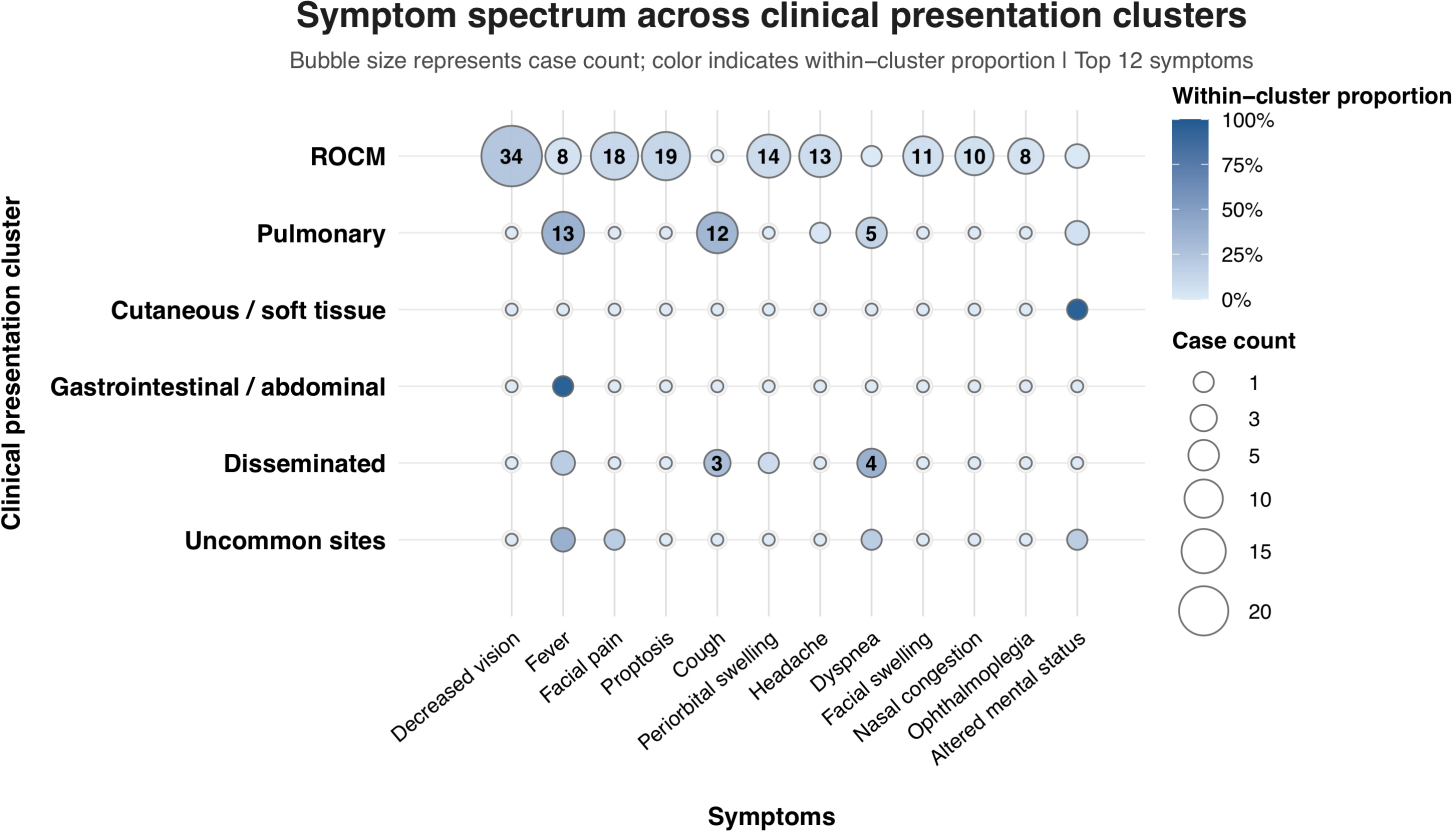
Structured symptom distribution patterns across clinical presentation phenotypes.

Distinct structural differences in symptom distribution were observed across clinical presentation clusters (**Figure 3**), characterized by a pattern of “intra-cluster aggregation and inter-cluster separation.” Specifically, relatively stable symptom combinations were identified within each cluster, whereas clearly distinguishable symptom patterns emerged between different clusters.

#### 3.4.1 Rhino-orbito-cerebral *mucormycosis*

The symptom distribution in ROCM was highly concentrated, predominantly involving orbital and craniofacial manifestations. The most common symptoms included vision loss (34 cases), proptosis (18 cases), and periorbital swelling (14 cases). Headache (13 cases) and facial swelling (11 cases) were also frequently observed, suggesting primary involvement of the paranasal sinuses and adjacent orbital structures.

Notably, although less frequent, ophthalmoplegia (10 cases) and altered mental status (8 cases) carried important clinical implications, potentially indicating intracranial extension of the infection.

Overall, ROCM demonstrated a highly aggregated symptom pattern centered on “visual impairment–orbital involvement–craniofacial manifestations,” reflecting a strong anatomical localization profile.

#### 3.4.2 Pulmonary *mucormycosis*

Pulmonary *mucormycosis* exhibited a symptom distribution pattern that was distinctly different from that of ROCM, with manifestations primarily confined to the respiratory system. Fever (13 cases) and cough (12 cases) were the predominant symptoms, while dyspnea (5 cases) was observed in a subset of patients, forming a typical infectious respiratory symptom complex.

In contrast to ROCM, pulmonary cases rarely presented with orbital or craniofacial symptoms, indicating a clear system-specific symptom profile. This distinction further reinforces the anatomical separation of symptom patterns across different clinical presentation clusters.

#### 3.4.3 Other clinical types (cutaneous/soft tissue, gastrointestinal, disseminated, and uncommon sites)

The remaining clinical types were characterized by smaller case numbers and more dispersed symptom distributions, without stable high-frequency symptom combinations. Their clinical manifestations were largely determined by the involved anatomical sites.

For example, cutaneous and soft tissue infections typically presented with localized lesions, gastrointestinal or abdominal infections manifested with digestive symptoms, and disseminated infections involved multiple organ systems, including neurological manifestations such as altered mental status.

Overall, these types exhibited a higher degree of heterogeneity in their symptom spectrum and lacked the concentrated distribution patterns observed in ROCM or pulmonary *mucormycosis*.

#### 3.4.4 Distribution of imaging features

Further analysis of imaging findings revealed a strong anatomical correlation across clinical presentation clusters (**Table 2**; **Supplementary Table 2**). ROCM was primarily characterized by sinus involvement, orbital invasion, and intracranial extension, suggesting disease progression from the paranasal sinuses to the orbit and central nervous system.

**Table 2:** Imaging features across clinical presentation clusters.

Clinical cluster	Key imaging features
ROCM	Sinus involvement; orbital invasion; intracranial extension
Pulmonary	Consolidation; nodules; cavitation
Cutaneous/soft tissue	Local soft tissue destruction
Gastrointestinal/abdominal	Abdominal lesions/nonspecific findings
Disseminated	Multi-organ involvement
Uncommon sites	Site-specific findings

In contrast, pulmonary *mucormycosis* was predominantly associated with imaging features such as pulmonary consolidation, nodules, or cavitary lesions. Imaging findings in other clinical types varied according to the anatomical site involved.

Overall, imaging characteristics were highly consistent with symptom distributions, further supporting the site-specific nature and structural coherence of different clinical presentation clusters.

### 3.5 Diagnostic pathway analysis

**Figure 4:**
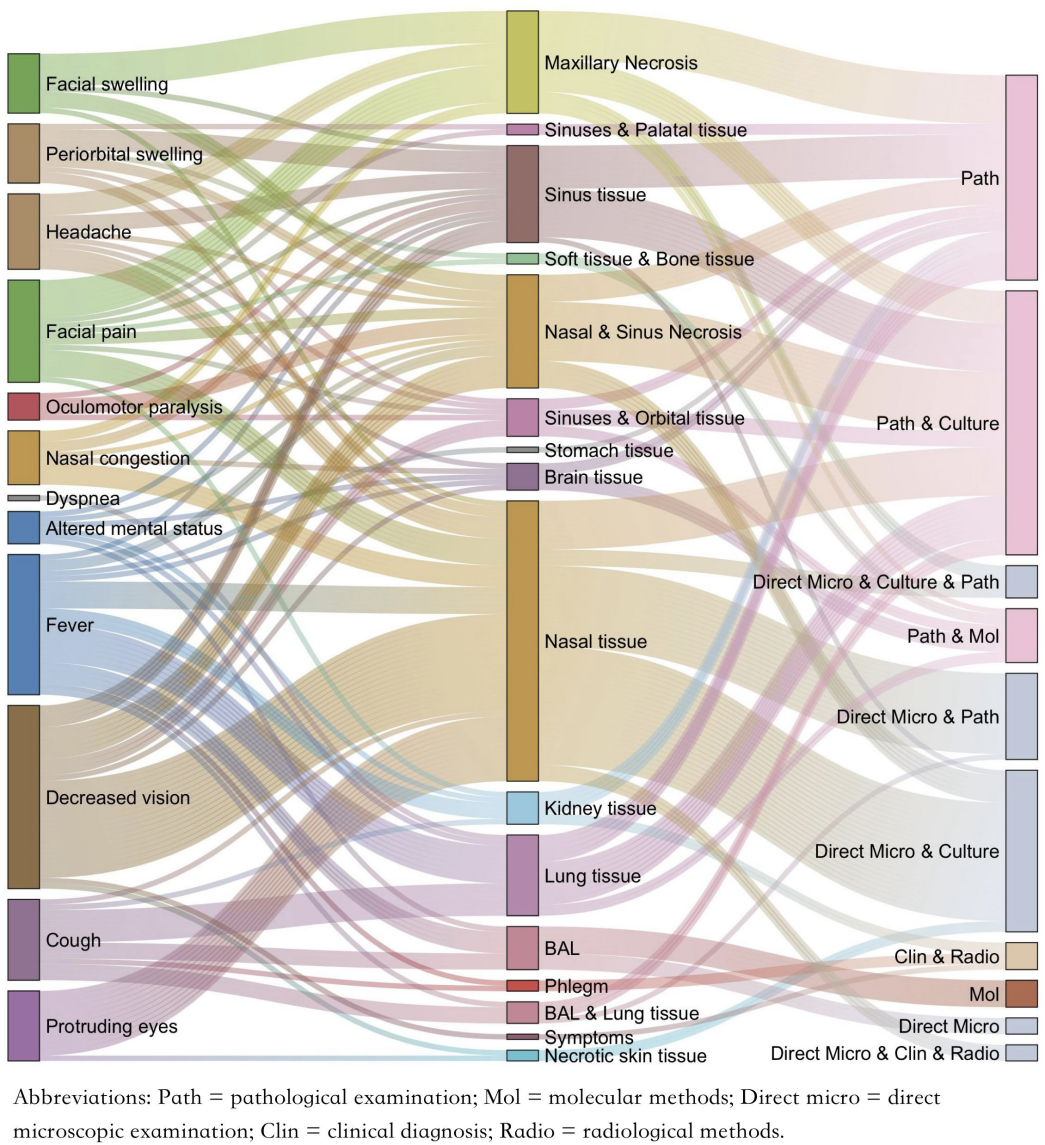
Evidence mapping of diagnostic pathways linking symptoms, sampling sites, and diagnostic methods.

Significant structural differences were observed across clinical presentation clusters in terms of sampling sites and diagnostic approaches, collectively forming a multi-level diagnostic pathway linking “clinical presentation–sampling site–diagnostic method” (**Figure 4**). Overall, this pathway exhibited a clear “symptom-driven” characteristic, whereby clinical manifestations directly influenced sampling strategies and subsequently determined the choice of diagnostic methods.

#### 3.5.1 Distribution of sampling sites

The distribution of sampling sites demonstrated a pronounced anatomical concentration. Tissues from the nasal cavity and paranasal sinuses were the predominant sources of specimens, including nasal tissue, sinus tissue, and necrotic material, accounting for the majority of cases.

Pulmonary specimens, including lung tissue and bronchoalveolar lavage (BAL) fluid, were the second most common sampling sources, primarily observed in pulmonary cases. In contrast, sampling from brain tissue, soft tissue, or renal tissue was relatively uncommon and was typically associated with advanced disease progression or infections involving uncommon anatomical sites.

These findings suggest that sampling strategies are strongly dependent on the anatomical location of infection.

#### 3.5.2 Distribution of diagnostic methods

Regarding diagnostic approaches, histopathological examination was the primary method for definitive diagnosis, with most cases relying on tissue-based pathology. In some cases, fungal culture or direct microscopic examination was performed in combination to enhance diagnostic reliability.

In contrast, exclusive reliance on molecular testing or imaging-based diagnosis was relatively uncommon. Overall, the combined use of multiple diagnostic modalities was frequently observed, reflecting the reliance on integrated evidence for the diagnosis of *mucormycosis*.

#### 3.5.3 Relationships between presentation clusters, sampling, and diagnosis

Further analysis revealed distinct stratification patterns in the combinations of sampling sites and diagnostic methods across clinical presentation clusters.

For ROCM cases, specimens were primarily obtained from the nasal cavity or paranasal sinuses, followed by histopathological examination, often supplemented by fungal culture. This resulted in a relatively concentrated diagnostic pathway characterized by “sinus-based sampling with pathology-dominant confirmation”.

In contrast, pulmonary cases were typically diagnosed through lung tissue or BAL sampling, combined with histopathological and microbiological methods, forming a pathway distinct from that of ROCM.

For other clinical types, due to the diversity of anatomical involvement, both sampling strategies and diagnostic pathways were more heterogeneous. Specimens could be obtained from brain tissue, soft tissue, or abdominal sites, and diagnostic method combinations were less standardized.

Overall, the diagnostic pathway of *mucormycosis* follows a structured pattern driven by clinical presentation, mediated by anatomical localization, and ultimately directed toward specific diagnostic methods. This “presentation–sampling–diagnosis” framework suggests that differences among clinical types extend beyond symptomatology to include systematic variations in diagnostic strategies.

### 3.6 Association between host factors and clinical presentation

Further analysis of the relationship between host factors and clinical presentation suggested potential associations between host status and the distribution of clinical presentation clusters. Notably, among patients with diabetic ketoacidosis (DKA), clinical manifestations appeared to be more concentrated in rhino-orbito-cerebral *mucormycosis*(ROCM), indicating that metabolic disturbances may be associated with the occurrence of this specific infection type.

In contrast, cases without DKA exhibited a broader range of anatomical involvement, including pulmonary and other less common forms, suggesting that different host conditions may be associated with distinct distribution patterns of *mucormycosis*.

It should be noted that DKA status and other host-related variables were not consistently reported across the included studies. Therefore, the above findings are based on descriptive observations and should not be interpreted as evidence of causal relationships.

Overall, host factors, particularly metabolic status, may play an important role in shaping the clinical presentation of *mucormycosis*; however, further studies are required to validate these associations.

## 4 Discussion

### 4.1 Principal findings and clinical implications

Based on a case-level evidence mapping analysis, this study systematically elucidated the structural characteristics of diabetic-associated *mucormycosis* across three key dimensions: distribution patterns, symptom composition, and diagnostic pathways.

First, rhino-orbito-cerebral *mucormycosis* (ROCM) was identified as the predominant clinical presentation, suggesting a marked anatomical predilection of this infection in diabetic hosts, which is consistent with previous studies (Cicek et al., 2026). Second, a distinct pattern of “intra-cluster aggregation and inter-cluster separation” was observed in symptom distribution. Specifically, ROCM was primarily characterized by visual and orbital manifestations, whereas pulmonary *mucormycosis* was dominated by respiratory symptoms (Qu et al., 2026).

In addition, the diagnostic pathway demonstrated a clear anatomical dependence, whereby clinical presentation directly influenced sampling strategies and the subsequent selection of diagnostic methods. Notably, conventional diagnostic approaches still present limitations in the context of early recognition (Qu et al., 2026).

Overall, this study constructed a structured clinical presentation framework for diabetic-associated *mucormycosis* from the integrated perspectives of “distribution–symptom–diagnostic pathway.” This framework provides a comprehensive and clinically relevant perspective that may facilitate early recognition and support optimization of diagnostic decision-making in high-risk populations. Therefore, the proposed framework should be interpreted as a descriptive and hypothesis-generating model intended to support conceptual understanding of clinical presentation patterns rather than a predictive or validated clinical tool.

### 4.2 Mechanistic links between diabetic host factors and infection phenotypes

Diabetes mellitus, particularly when complicated by diabetic ketoacidosis (DKA), is widely recognized as a major host predisposition for *mucormycosis*. In the present study, the predominance of rhino-orbito-cerebral *mucormycosis* (ROCM) suggests that host metabolic abnormalities may drive the selective distribution of infection phenotypes through specific biological mechanisms.

Previous studies have demonstrated that hyperglycemia and an acidic environment impair neutrophil chemotaxis and phagocytic function, while simultaneously promoting the release of free iron in serum, thereby creating favorable conditions for fungal growth and invasion. In addition, *mucormycosis* is characterized by pronounced angioinvasion, which can lead to thrombosis, tissue ischemia, and extensive necrosis—processes considered central to its high mortality (Cicek et al., 2026; Qu et al., 2026).

Notably, these pathological processes appear to be particularly prominent in the paranasal sinus and rhino-orbital-cerebral regions. The rich vascular network and anatomical continuity in these areas may provide a “low-resistance pathway” for fungal dissemination, facilitating extension from the sinuses to the orbit and central nervous system. This mechanism may partially explain the predominance of ROCM observed in this study.

In contrast, in the absence of DKA or under different host immune conditions, the anatomical distribution of infection appears more heterogeneous, including pulmonary and other less common forms. This observation suggests that host metabolic status may not only influence the risk of infection but also contribute to determining its anatomical localization and clinical phenotype.

Overall, the sequence of “hyperglycemia/acidosis–altered iron metabolism–angioinvasion–tissue necrosis” may be conceptualized as a key pathophysiological axis underlying diabetic-associated *mucormycosis*. This mechanistic cascade provides a plausible explanation for the predominance of ROCM and offers a framework for understanding phenotypic variability across different clinical presentations.

### 4.3 From symptoms to recognition patterns: a structured interpretation of the clinical presentation spectrum

One of the central contributions of this study lies in shifting the descriptive interpretation of diabetes-associated mucormycosis from isolated symptoms toward a pattern-based perspective centered on symptom combinations. The evidence mapping findings suggest that clinical features may not occur entirely independently but instead appear to form relatively stable “presentation cluster–symptom combination” structures associated with specific anatomical sites of infection. Such clustered manifestations may not be entirely incidental, but may instead reflect the underlying anatomical progression and invasive nature of mucormycosis.

These clustered symptom patterns may also reflect the angioinvasive and anatomically progressive nature of mucormycosis. In ROCM, fungal invasion often originates in the paranasal sinuses and subsequently extends along the sinus–orbital–intracranial axis through angioinvasive extension involving vascular invasion and adjacent tissue spread. This progression may explain the characteristic aggregation of orbital and craniofacial manifestations, including periorbital swelling, ophthalmoplegia, proptosis, and vision loss. As tissue invasion advances, neurological manifestations such as altered mental status may emerge, potentially indicating intracranial extension and advanced disease severity (Cicek et al., 2026; Qu et al., 2026).

In contrast, pulmonary mucormycosis primarily involves invasion of the lower respiratory tract and pulmonary vasculature, often accompanied by vascular thrombosis and tissue necrosis, which may contribute to relatively concentrated respiratory symptom patterns such as fever, cough, and dyspnea (Qu et al., 2026). These findings suggest that the identified symptom clusters may not simply represent co-occurring manifestations, but may also partially reflect structured anatomical progression and site-specific invasive behavior in diabetes-associated mucormycosis.

Previous literature has largely emphasized the rapid progression, high mortality, and clinical complexity of mucormycosis (Qu et al., 2026). Yet systematic integration of recognizable presentation patterns across different clinical types has remained limited. In the present descriptive synthesis, ROCM was characterized not only by frequent ocular manifestations but also by a concentrated symptom combination involving vision loss, proptosis, periorbital swelling, headache, facial swelling, and ophthalmoplegia.

Similarly, pulmonary mucormycosis demonstrated a relatively distinct respiratory symptom profile, including fever, cough, and dyspnea, whereas other clinical types, likely due to their more heterogeneous anatomical involvement, lacked stable high-frequency symptom combinations. The observed pattern of “intra-cluster aggregation and inter-cluster separation” may suggest that mucormycosis in diabetic hosts exhibits certain recognizable phenotypic structures within the published literature.

From a clinical perspective, delayed diagnosis of mucormycosis may partly result from the overlap between its early manifestations and those of more common infectious conditions, such as sinusitis, orbital cellulitis, or pulmonary infections (Sharma and Goel, 2022). In this context, recognition of symptom combinations rather than isolated manifestations may contribute to a more structured conceptual understanding of clinical presentation patterns in high-risk populations.

For example, in diabetic patients—particularly those with poor glycemic control or DKA—the coexistence of visual disturbances, orbital signs, and craniofacial symptoms may warrant increased clinical attention to the possibility of ROCM. Likewise, in high-risk hosts presenting with fever, cough, and dyspnea accompanied by invasive radiological findings, pulmonary mucormycosis may be considered within the differential diagnostic context. Previous studies have suggested that acidic conditions and altered iron metabolism in diabetic ketoacidosis may facilitate fungal proliferation and tissue invasion (Ibrahim, 2011).

**Figure 5:**
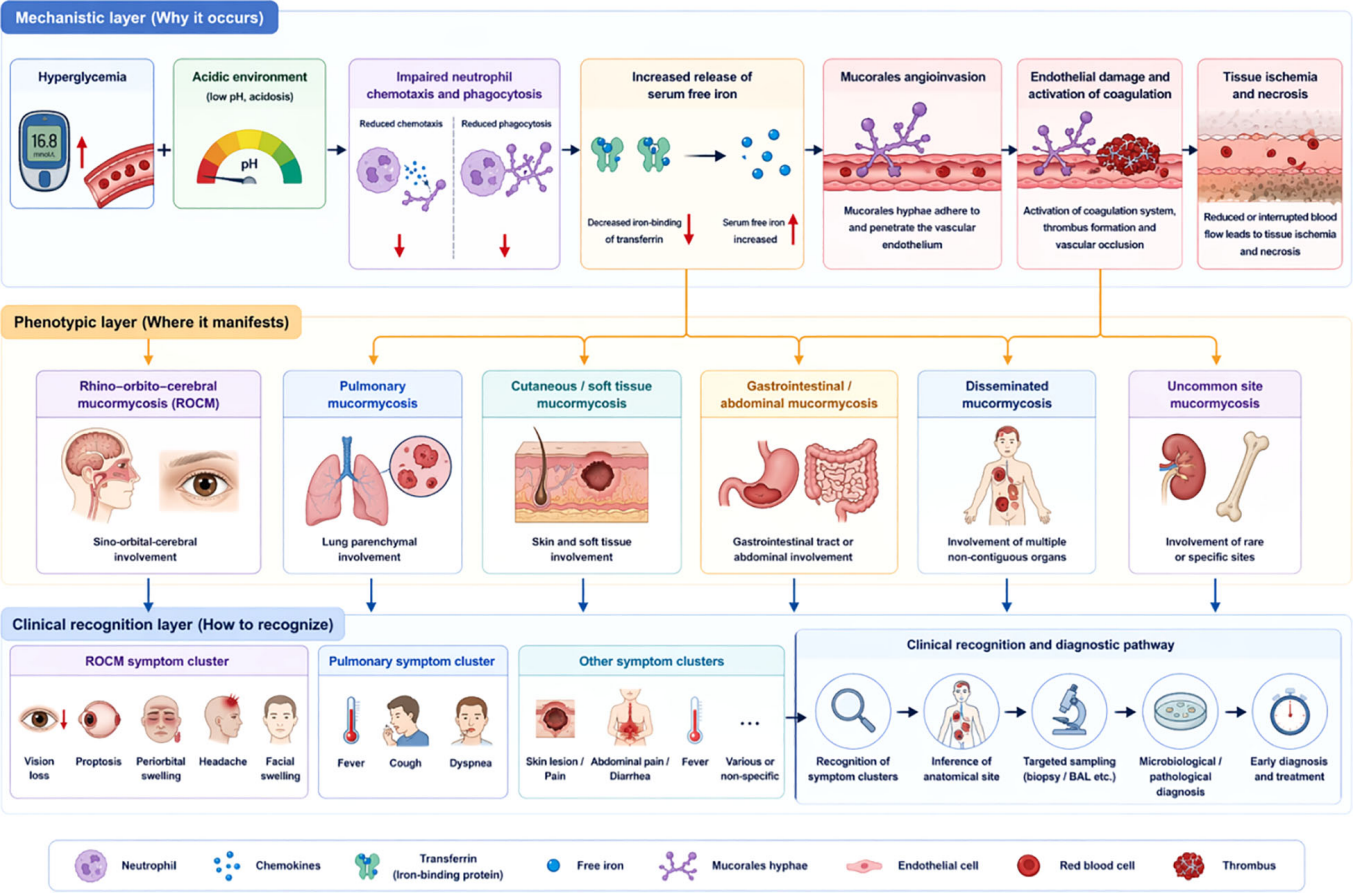
Schematic illustration of the proposed mechanism–phenotype–recognition framework in diabetes-associated *mucormycosis*, synthesized from existing pathophysiological literature and the present evidence mapping findings.

Therefore, the framework proposed in this study should be interpreted as a descriptive and hypothesis-generating recognition model rather than a validated predictive clinical tool. The conceptual transition from “symptom enumeration” to “pattern recognition” may help inform future studies exploring clinical recognition pathways and diagnostic strategies in diabetes-associated mucormycosis (**Figure 5**). This pattern-based perspective may be particularly relevant in diabetic hosts, in whom delayed recognition can rapidly lead to extensive tissue invasion and poor clinical outcomes.

### 4.4 Clinical implications and optimization of diagnostic pathways

In contrast to the structured recognition of clinical presentations, the key challenge in diagnostic pathways lies in translating recognition patterns into actionable clinical decisions. The findings of this study suggest that, in the context of diabetes as a high-risk host condition, the diagnostic process of mucormycosis may follow a clinically structured recognition-to-sampling pathway.

The observed relationships between presentation patterns and diagnostic pathways may reflect how clinicians adapt diagnostic strategies according to anatomical localization and disease severity. In ROCM, clustered craniofacial and orbital manifestations may prompt earlier suspicion of invasive fungal infection and facilitate targeted sampling from the nasal cavity or paranasal sinuses. In contrast, pulmonary mucormycosis often presents with nonspecific respiratory symptoms that substantially overlap with bacterial pneumonia, tuberculosis, or other pulmonary infections. Because early manifestations are frequently nonspecific and may initially mimic more common infectious conditions, pulmonary cases may be particularly vulnerable to delayed recognition and delayed acquisition of invasive respiratory specimens (Sharma and Goel, 2022; Qu et al., 2026).

Moreover, because mucormycosis is characterized by rapid angioinvasive progression and tissue necrosis, delays in targeted sampling and etiological confirmation may allow continued extension of infection toward orbital or intracranial structures. Therefore, the clinical relevance of symptom-pattern recognition may extend beyond descriptive classification and may contribute to earlier diagnostic decision-making and anatomically directed sampling strategies in high-risk diabetic hosts.

In clinical practice, when diabetic patients present with features suggestive of rhino-orbito-cerebral mucormycosis (ROCM), management based solely on conventional sinusitis protocols may be insufficient to address the possibility of invasive fungal infection. In such cases, early sampling from the nasal cavity or paranasal sinuses, combined with histopathological examination, should be considered to improve diagnostic accuracy.

Similarly, in patients with predominantly respiratory symptoms—particularly when imaging findings indicate invasive disease—specimen collection via bronchoalveolar lavage (BAL) or lung tissue sampling may be prioritized to support diagnostic evaluation.

Importantly, optimization of the diagnostic pathway does not primarily depend on increasing the number of diagnostic modalities, but rather on appropriately advancing the timing of targeted sampling. Previous studies have demonstrated that poor outcomes in mucormycosis are closely associated with delays in diagnosis (Ibrahim et al., 2008; Gulmez et al., 2025; Naddafard et al., 2025; Rabagliati et al., 2025; Wan et al., 2025; Cicek et al., 2026). Therefore, in diabetic patients, when clinical features suggest a specific site of infection, early targeted sampling and prompt etiological evaluation may help shorten the time to diagnosis and reduce delays in management. This may be particularly important in ROCM, where delayed diagnosis can rapidly lead to orbital invasion, intracranial extension, and irreversible tissue damage.

### 4.5 Evidence gaps and future research directions

Despite the systematic integration of the clinical presentation spectrum and diagnostic pathways of diabetic-associated *mucormycosis* in this study, several important evidence gaps remain.

First, host-related information was incompletely reported across the included studies. In particular, key metabolic variables such as diabetic ketoacidosis (DKA) were missing in a substantial proportion of cases, limiting in-depth analysis of the relationship between host factors and clinical phenotypes.

Second, the reporting of symptoms and imaging findings lacked standardization, resulting in considerable heterogeneity across studies (**Figure 3**), which may have affected the refinement and precision of the constructed clinical presentation spectrum.

Third, the current evidence base is predominantly derived from case reports, with an imbalanced distribution of clinical types, largely dominated by rhino-orbito-cerebral *mucormycosis* (ROCM), while other forms remain underrepresented.

Future research should prioritize standardized reporting of clinical and host-related variables and adopt more systematic data collection approaches. In particular, multicenter cohort studies and large-scale database analyses are needed to capture a broader spectrum of clinical types. Such efforts would enable further validation and refinement of the pattern-based clinical recognition framework proposed in this study.

### 4.6 Limitations

Several limitations of this study should be acknowledged.

First, the included evidence was predominantly derived from case reports and case series, which may introduce selection bias. In addition, the distribution of clinical types was imbalanced, with a predominance of rhino-orbito-cerebral *mucormycosis* (ROCM), which may have led to an overestimation of its relative proportion and potentially affected the representativeness of the overall disease spectrum.

Second, key host-related variables (e.g., diabetic ketoacidosis [DKA] status) and imaging data were incompletely reported in the original studies. This limitation restricted in-depth analysis of the relationship between host factors and clinical phenotypes and may have resulted in an underestimation of differences across clinical presentation types.

Third, the lack of standardized reporting of symptoms and imaging findings across studies introduced heterogeneity during data integration, which may have affected the granularity of the constructed clinical presentation spectrum.

Fourth, as this study employed an evidence mapping approach with descriptive synthesis, no quantitative analysis or causal inference was performed. Therefore, the findings are primarily intended to describe distribution patterns and identify potential structures, rather than to support risk estimation or effect size quantification. This methodological choice is consistent with the study objectives, which focused on evidence distribution and structured pattern recognition rather than causal inference.

Finally, as this study was based on published literature, publication bias is unavoidable. In particular, typical or severe cases are more likely to be reported, which may influence the generalizability of the findings.

Despite these limitations, this study provides a systematic case-level synthesis and, for the first time, constructs a structured clinical presentation framework for diabetic-associated *mucormycosis* across three dimensions—distribution, symptoms, and diagnostic pathways. In the context of a fragmented evidence base dominated by case reports, this integrated perspective offers meaningful insights for understanding disease patterns and optimizing early clinical recognition strategies. Given that the present study was based predominantly on published case reports and case series, the identified distribution patterns and clinical presentation structures should be interpreted as descriptive representations of the published literature rather than estimates of the true epidemiological distribution of diabetic-associated *mucormycosis*.

## 5 Conclusions

This study, based on an evidence mapping approach, identified the structural characteristics of diabetic-associated *mucormycosis* across distribution patterns, symptom profiles, and diagnostic pathways. The findings indicate that rhino-orbito-cerebral *mucormycosis* (ROCM) is the predominant clinical form, and that symptom patterns exhibit a distinct structure characterized by “intra-cluster aggregation and inter-cluster separation.” In addition, a diagnostic pathway driven by clinical presentation was observed.

Building on these findings, we propose a structured recognition framework linking “presentation clusters–symptom combinations–diagnostic pathways.” This framework provides a structured descriptive synthesis linking presentation clusters, symptom combinations, and diagnostic pathways in diabetic-associated *mucormycosis*. The findings may serve as a hypothesis-generating basis for future studies exploring clinical recognition patterns and diagnostic strategies in high-risk populations.
