# Supplementary material for: Pattern-based clinical recognition of diabetes-associated mucormycosis: an evidence mapping study integrating symptom clustering and diagnostic pathways
Source: Front Cell Infect Microbiol. 2026 Jul 3;16:1861349. doi: 10.3389/fcimb.2026.1861349 (PMC13375622; doi:10.3389/fcimb.2026.1861349)
Supplement: Supplementary file 1 [file Table1.docx]

| Supplementary Table 1. Search strategy | | |
| --- | --- | --- |
| Database | Search strategy | Results |
| PubMed | (  "Mucormycosis"[Mesh]   OR mucormycosis[tiab]   OR zygomycosis[tiab]   OR mucorales[tiab]  OR rhizopus[tiab]  OR mucor[tiab]  OR lichtheimia[tiab] ) AND (  "Diabetes Mellitus"[Mesh]   OR diabetes[tiab]   OR diabetic[tiab]   OR "diabetic ketoacidosis"[tiab]   OR DKA[tiab] ) AND (  "Signs and Symptoms"[Mesh]  OR "Clinical Features"[tiab]  OR "clinical presentation"[tiab]  OR symptoms[tiab]  OR imaging[tiab]  OR diagnosis[tiab]  OR diagnostic[tiab]  OR confirmed[tiab]  OR proven[tiab]  OR probable[tiab] ) | 1,517 |
| Embase | ('mucormycosis'/exp OR mucormycosis:ti,ab OR mucorales:ti,ab) AND ('diabetes mellitus'/exp OR diabetic*:ti,ab OR 'diabetic ketoacidosis':ti,ab) AND ('clinical feature'/exp OR 'clinical presentation':ti,ab OR diagnos*:ti,ab) NOT ('conference abstract'/it OR 'conference paper'/it) AND [humans]/lim AND [english]/lim | 1,268 |
| Web of Science | (mucormycosis OR mucorales) AND (diabetes OR diabetic OR "diabetic ketoacidosis") AND (clinical OR presentation OR diagnosis OR diagnostic) | 1463 |
